# Associations of serum C-peptide and insulin-like growth factor binding proteins-3 with breast cancer deaths

**DOI:** 10.1371/journal.pone.0242310

**Published:** 2020-11-12

**Authors:** PinYu Cui, Yuan Chen, Nuremaguli Waili, YaXing Li, CuiLing Ma, Ying Li

**Affiliations:** Department of Public Health, School of Medicine, Zhejiang University, Hangzhou, China; San Raffaele Roma Open University, ITALY

## Abstract

C-peptide is usually considered as a marker of insulin secretion and has no physiological function. This study aimed to assess the association between serum C-peptide level as independent risk factor and breast cancer and explored the possible underlying mechanisms. This was a population-based cohort study. All the data was collected according to a standard protocol. The C-peptide and insulin-like growth factor binding proteins-3(IGFBP-3) concentrations were measured in blood. The breast cancer deaths were confirmed by National Death Index records. Cox proportional hazard regression analysis was conducted to determine the hazard ratio of serum C-peptide level for breast cancer deaths. Analysis of covariance was used to assess the association between serum C-peptide and IGFBP-3 level, and the linear trend was tested by using a linear model. A total of 8,373 women 17 years of age or older were included in the study, and 57 breast cancer deaths were observed over the study period. The result of survival analysis showed that breast cancer deaths increased with increasing levels of serum C-peptide. The hazard ratio was 1.69 (95% confidence interval, 1.17–2.45). The levels of circulating IGFBP-3 were positively associated with changes in serum C-peptide levels and showed a strong linear trend in the covariance analysis. Serum C-peptide level was associated with increased risk of breast cancer death. Our results suggest that the increased risk of breast cancer death can be via a pathway that serum C-peptide level positive associated with the change in serum IGFBP-3 level.

## Introduction

Breast cancer is the most common malignancy and the second leading cause of death among women worldwide. Moreover, the incidence of and mortality related to breast cancer keep rising in most countries despite efforts to prevent cancer in the past 20 years. More than one million women are diagnosed with breast cancer annually, and the economic burden of disease continues to increase globally. Therefore, breast cancer remains a major public health challenge for women worldwide [1, 2].

The exact cause of breast cancer has not been completely elucidated. However, previous studies have identified several risk factors that increase the risk of breast cancer events and deaths in women, including modifiable and non-modifiable risk factors. Non-modifiable risk factors include age, family history of breast cancer, and genetic factors. Modifiable risk factors include lifestyle-related factors, such as smoking, alcohol consumption, physically inactivity, radiation exposure, and drug abuse. Moreover, risk factors that are called potentially modifiable risk factors, such as age of menarche, age of menopause, few pregnancies, and short or no periods of breastfeeding have been identified [3]. Recent studies have shown that the breast cancer type 1 susceptibility protein gene (BRCA1) and breast cancer type 2 susceptibility protein gene (BRCA2) germline mutations are major causes for cancer predisposition in families with a history of breast cancer. Studies have estimated that the penetrance of BRCA1 and BRCA2 gene mutations are association with the incidence of breast cancer only in 6%–8% of all breast cancer cases, and the cumulative incidence of sporadic breast cancer in approximately 90%–95% of the general population [4, 5]. Great progress has been made in tracing the etiology and prevention study of breast cancer over the last several decades, but results have showed that breast cancer incidence has not decreased as intended; moreover, it has shown an increasing trend in many countries. The increase in incidence of breast cancer can be attributed to an increase in women with major breast cancer risk factors, such as low age of menarche, obesity, changes in serum hormone levels, or increased screening [6]. Additionally, other studies have shown that the impact of hereditary breast cancer has increased. The major cause is estimated to be the penetrance of the BRCA1 and BRCA2 founder mutation having increased over the last century, as observed by the Collaborative Group on Hormonal Factors in Breast Cancer [7, 8]. The etiology of breast cancer is complex and multifactorial, and the related risk factors may be directly causative or may enhance the risk in the presence of the causative risk; moreover, the related risk factors may be secondary manifestations of basic underlying metabolic abnormalities, among which belong cellular pathways that involve cell growth and proliferation, such as the MAPK, P13K/AKT/mTOR, and TP53 pathways [9, 10]. However, increased that those were found risk factors impact on breast cancer and the mechanism of direct and indirect is poorly understood.

Numerous epidemiologic studies have shown that the serum C-peptide level is independently associated with cardiovascular disease, and cancer and total mortality [11]. Moreover, serum C-peptide level has been associated with increased breast cancer risk in several studies [12, 13]. In the past, the role of C-peptide in human physiology has not been clarified. As a marker of insulin secretion, the insulin secretion rate is usually determined by serum C-peptide level. In recent years, evidence based on clinical and experimental studies have suggested that C-peptide is an active peptide hormone with an important physiologic function. It might be involved in glucose transport and microvascular blood flow stimulation. Additionally, serum C-peptide level is strongly associated with body fat distribution and is independent of serum insulin and fasting plasma glucose level. Furthermore, serum C-peptide level is significantly elevated in patients with metabolic syndromes and diabetes. C-peptide has been shown to participate in the anti-apoptotic process of TNF-alpha by activating several key-signaling molecules, such as PKC and MAP kinase family members [14].

However, the etiology of breast cancer is complex and unclear. Studies have reported the association between serum C-peptide level and breast cancer, but several important questions have remained unanswered. First, previous studies did not assess whether the association between serum C-peptide levels and breast cancer is independent of serum insulin level but only as an indicator of insulin secretion [15]. Second, these studies have failed to report a possible etiological mechanism. Third, some important confounding factors were poorly controlled. Serum C-peptide level may be changed by healthy diet and regular physical activity. Therefore, the objective of this study is to identify the association between serum C-peptide levels and the risk of breast cancer after adjusting for a large number of potential risk factors and to discuss the possible pathogenesis.

## Materials and methods

### Study population

**Fig 1:**
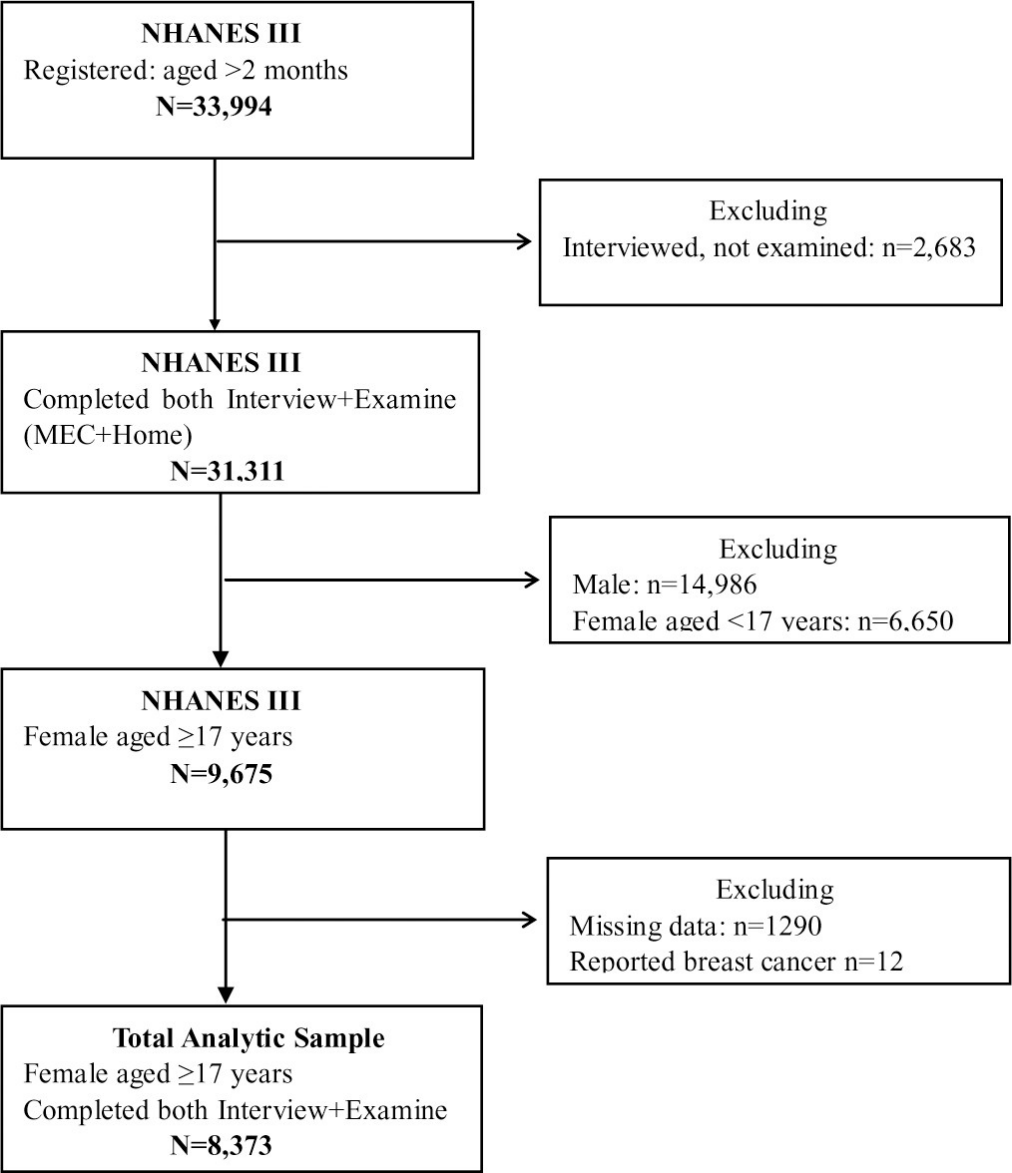
Flowchart of participant selection at baseline. Among 33,944 participants who were interviewed in NHANES III, 31,311 participants completed questionnaire and received physical examinations at the mobile examination center and home. In the present analysis, we included 8,373 females aged ≥17 years who reported no history of breast cancer.

This study was based on data from the National Health and Nutrition Examination Survey III (NHANES III), a national cohort survey that aimed to assess the health and nutritional status in the general population of the United States. Baseline data were collected from 1988 to 1994 using a complex, multistage cluster sampling design. The survey was divided into 2 phases; the first phase was conducted from 1988 to 1991 and surveyed 44 locations, and the second phase was conducted from 1991 to 1994 and surveyed 45 locations. A representative sample comprising 33,994 US residents aged >2 months was registered. The 33,994 US residents were interviewed and 30,818 were examined in the 6-year sample. Breast cancer deaths were ascertained by linking the records to National Death Index records. Participants were included in this study if they met the following selection criteria (Fig 1): women aged ≥17 years old with available mortality follow-up data and had completed interviews and physical examinations at the mobile examination center (MEC) and home. We excluded subjects whose serum C-peptide measurement values were unavailable or missing. Participants who self-reported previous breast cancer history also were excluded from the study. Other details about the survey are described elsewhere. All participants provided written informed consent. The study was approved by the institutional review board of the Center for Disease Control and Prevention (Atlanta, Georgia, USA).

### Data collection

The NHANES III survey data consisted of 3 parts: home interview, clinical tests, and physical examinations. The home interview included participants’ self-reported demographics, behavior habits, and general health statuses. Participants were asked whether they had engaged leisure time physical activities and specific exercises in the past month, and the frequency and intensity of each activity were also recorded. Smoking status was categorized as never, former, or current smoker. Tobacco use was assessed by the age of initiation, frequency, and total number of years for which they had or have been smoked. Alcohol intake was measured by recalling the frequency, amount, and type of alcoholic beverages consumed. Participants were defined as drinkers if they had consumed at least 12 drinks during the past 12 months. Total daily energy intake consumed from fat, carbohydrates, and protein were obtained from the 24-hour dietary recall at the MEC.

The body measurements involved measuring the weight, height, lengths, skinfolds, circumferences, and breadths. Body Mass Index (BMI) was calculated as the weight in kilograms divided by the square of the height in meters (kg/m2). Waist circumference (WC) was measured by using a metal tape. Waist-to-hip ratio was calculated as waist circumference divided by the circumference buttocks. Skinfold thickness was measured at four different anatomic body sites.

Blood and urine specimens were obtained, and several tests and measurements were performed following laboratory procedures used for the NHANES III. Serum C-peptide, serum insulin, and fasting plasma glucose levels were measured in participants who had fasted for 8 to 24 hours during the morning examination session. The serum concentrations of insulin-like growth factor-1(IGF-1) and insulin-like growth factor binding proteins-3 (IGFBP-3) were determined by using IGF-1 enzyme-linked immunosorbent assay and IGFBP-3 immunoradiometric assay.

### Statistical analysis

The demographic characteristics of study participants were analyzed by using descriptive statistics. The weighted percentages for general population and breast cancer cases were calculated by categorical variables according to the analytic and reporting guidelines for the NHANES III.

Cox proportional hazards model was used to identify the association between serum C-peptide levels and breast cancer death. The hazard ratios (HRs) and 95% confidence intervals (CIs) were computed to obtain serum C-peptide levels, serum insulin levels, and plasma glucose levels. The model 1 adjusted following variables: age, ethnicity, smoking status, alcohol use, education levels, physical activity, BMI, serum creatinine level, and menopausal status. The model 2 included serum C-peptide levels, serum insulin levels, and plasma glucose levels by using the stepwise method.

We conducted analysis of covariance (ANCOVA) to evaluate the association among the changes in IGFBP-3, serum C-peptide, and serum insulin levels. The serum IGFBP-3 level was the dependent variable, and serum C-peptide and serum insulin levels were the independent variables. The serum C-peptide levels were divided into four categories based on quartiles. The first quartile was defined as the 25th percentile (0.40 nmol/L), the median value represented the 50th percentile (0.65 nmol/L), and the third quartile was defined as the 75th percentile (1.00 nmol/L). The serum insulin levels were divided into four categories by the 25th percentile (39.12 pmol/L), 50th percentile (56.34 pmol/L), and 75th percentile (86.10 pmol/L) using the same method. Comparisons were made among four categories by using an F test with a significance level of 0.05, and the linear trend was assessed by using a general linear model. The first quartile was used as the reference group. The mean and standard error were calculated for serum IGFBP-3 levels by using the quartiles of serum C-peptide levels and serum insulin levels. The ANCOVA covariates included age, ethnicity, smoking status, alcohol use, education levels, physical activity, and BMI. Normality test and variance homogeneity test were carried out for every dependent variable. Further analyses were performed to evaluate the changes in the subscapular skinfold (SSF) and WC in the quartiles of the serum C-peptide levels after stratifying for insulin levels (<56.34 pmol/L, and ≥56.34 pmol/L).

We plotted survival functions for two groups with different levels of serum C-peptide (<1.002 nmol/L, and ≥1.002 nmol/L) by using the Kaplan-Meier method and made comparisons across breast cancer deaths among groups using the log-rank test. All analyses were performed by using SAS 9.2 for Windows (SAS Institute. Cary, NC, USA).

## Result

The characteristics of the study participants are shown in Table 1. A total of 8,373 women aged ≥17 years and 57 breast cancer deaths were reported during the study period. In the present study, the lowest mortality rate was observed in the age group under 30 years old, and 45.6% of breast cancer deaths occurred in women over 60 years old. Moreover, 41.9% of the participants were non-Hispanic white women, but the mortality rate of breast cancer was highest in Non-Hispanic black women. Additionally, 33.3% of women with breast cancer has less than nine years of education. The participants who reported they were former smokers had higher breast cancer deaths than those who reported they were never-smokers. Furthermore, 52.6% of breast cancer cases had BMI values greater than or equal to 30 kg/m^2^.

**Table 1:** Baseline characteristics of participants in the study.

Variable	Control		Case		*P* Value	
	(n = 8,316) (%)		(n = 57) (%)			
Characteristic variables (N, %)						
Age groups (yr)						
< 30	1757	(21.1)	3	(5.3)	0.008	
30−44	2533	(30.5)	15	(26.3)		
45−59	1463	(17.6)	13	(22.8)		
≥60	2563	(30.8)	26	(45.6)		
Race-ethnicity						
Non-Hispanic white	3486	(41.9)	24	(42.1)	0.571	
Non-Hispanic black	2331	(28.0)	20	(35.1)		
Mexican-American	2144	(25.8)	11	(19.3)		
Other	355	(4.3)	2	(3.5)		
Education (yr)						
0−8	1848	(22.2)	19	(33.3)	0.249	
9−11	1346	(16.2)	8	(14.1)		
12	2761	(33.2)	17	(29.8)		
≥13	2361	(28.4)	13	(22.8)		
Physical activity						
Yes	3131	(37.6)	29	(50.9)	0.040	
No	5158	(62.4)	28	(49.1)		
Smoking status						
Never	5012	(60.3)	30	(52.6)	0.077	
Former	1888	(22.7)	20	(35.1)		
Current	1416	(17.0)	7	(12.3)		
Alcohol use						
Yes	5691	(68.4)	42	(73.7)	0.369	
No	2625	(31.6)	15	(26.3)		
Body mass index (kg/cm^2^)						
<30	5805	(69.8)	30	(52.6)	0.021	
≥30	2511	(30.2)	27	(47.4)		
Measured Variables (Mean, SD)						
Serum C-peptide (nmol/L)	5.6	(2.2)	6.4	(3.4)	0.002	
Serum insulin (pmol/L)	78.8	(13.9)	93.3	(16.1)	0.376	
Plasma glucose (mmol/L)	101.1	(38.9)	116.8	(61.8)	0.060	
Subscapular skinfold (mm)	22.1	(10.3)	24.0	(10.5)	0.172	
Waist circumference (cm)	91.5	(15.1)	100.4	(16.6)	0.001	
Serum creatinine (umol/L)	86.7	(20.8)	88.6	(18.6)	0.655	

The results of time-to-event analysis by Cox proportional hazard regression model are shown in Table 2. A total of 1,349,680-person months of follow-up were calculated from the MEC date, and we observed 57 breast cancer deaths. In model 1, the increased serum C-peptide level and plasma glucose level were significantly associated with breast cancer deaths after adjustment for important confounding factors. The HRs were 1.69 (95% CI, 1.17–2.45) and 1.10 (95% CI, 1.02–1.18). The association between serum insulin level and breast cancer deaths was not statistically significant. In model 2, the plasma glucose level and serum insulin level were not associated with increased the risk of breast cancer deaths, but serum C-peptide level remained statistically significant.

**Table 2:** The hazard ratios of serum C-peptide levels for deaths of breast cancer in the study.

	Hazard ratio	95% Confidence interval		*P* Value	
Serum C-peptide levels (nmol/L)					
Model 1	1.69	1.17	2.45		0.005
Model 2	1.68	1.02	2.76		0.041
Serum insulin levels (pmol/L)					
Model 1	1.00	0.99	1.00		0.317
Model 2	0.99	0.99	1.00		0.656
Plasma glucose levels (mmol/L)					
Model 1	1.09	1.02	1.18		0.009
Model 2	1.00	0.99	1.00		0.895

Adjusted for age, ethnicity, education levels, physical activity, smoking status, alcohol use, body mass index, serum creatinine and menopausal status.

Model 2 included serum levels of C-peptide and insulin, plasma glucose levels.

**Fig 2:**
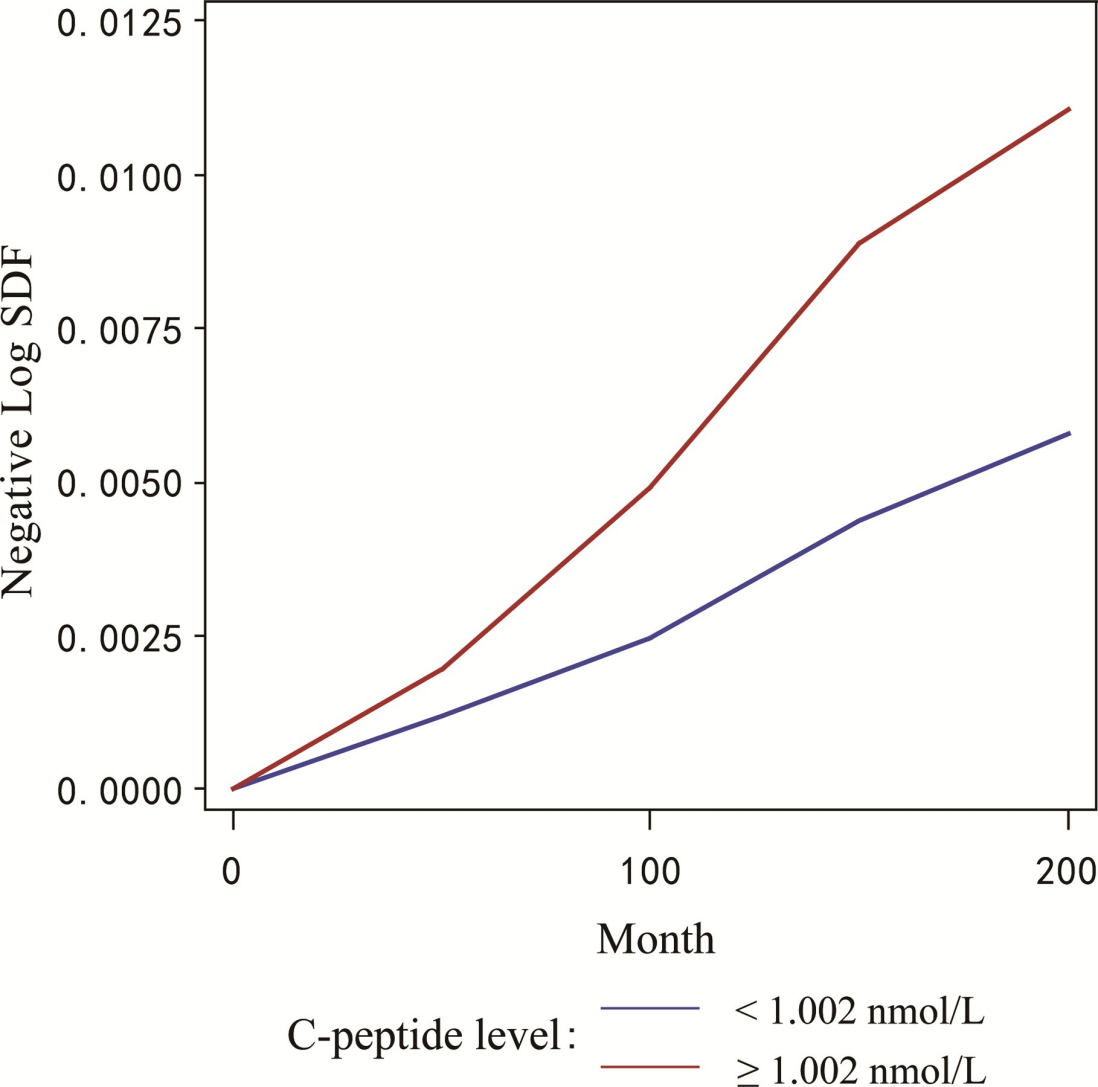
The cumulative deaths of breast cancer in the group with different levels of serum C-peptide. The negative log transformed survival function estimated the probability of breast cancer death in two groups with different serum C-peptide levels using Cox proportional hazards model. The Y-axis represents the negative log transformed survival function, while the X-axis shows the time in months.

The negative log transformed survival function estimated the hazard ratio of breast cancer in two groups with different serum C-peptide levels over the study period. A higher cumulative incidence of breast cancer deaths was observed in the group with higher serum C-peptide level compared with the group with lower serum C-peptide levels (Fig 2).

**Fig 3:**
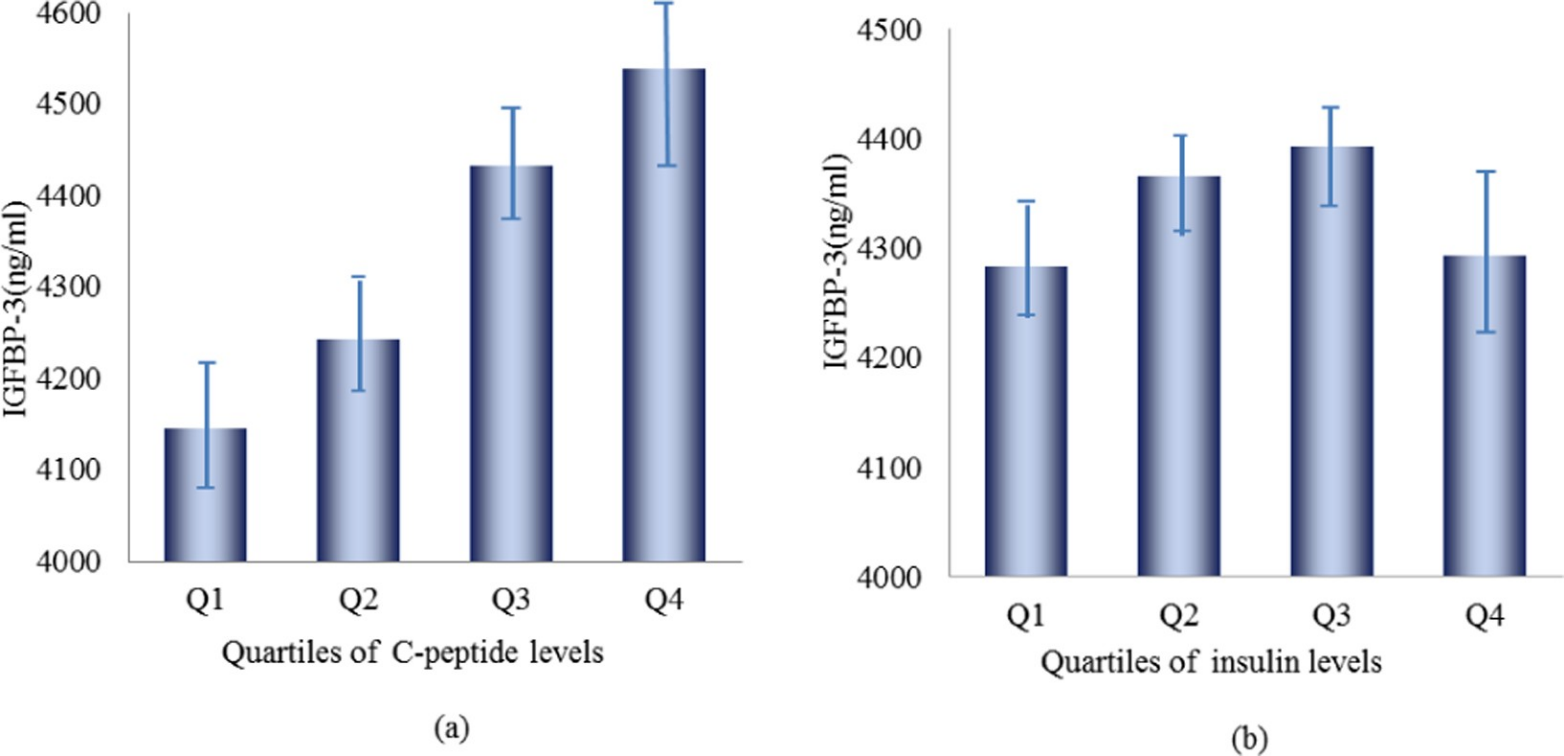
The means of serum IGFBP-3 levels in different quartiles of serum C-peptide and insulin. (a) and (b) showed that the means of serum IGFBP-3 levels in different quartiles of serum C-peptide and insulin adjusted for age, ethnicity, smoking status, alcohol use, education levels, physical activity and body mass index.

Table 3 and Fig 3 shows the changes in mean serum IGFBP-3 levels between the different quartiles of serum C-peptide levels. Compared with the reference group (25th percentile), the 25–50th percentile group, 50–75th percentile group, and 75th percentile group had gradually higher serum IGFBP-3 levels, which resulted in a significant linear trend. By contrast, the levels of serum IGFBP-3 were not associated with the change in levels of serum insulin based from the result of ANCOVA.

**Table 3:** The change of serum IGFBP-3 levels in different quartiles of serum C-peptide and insulin by analysis of covariance.

Variables	Levels of IGFBP-3 ^a^ (ng/mL)		
	Mean	SE^b^	*Р* Value
Serum C-peptide (nmol/L)			
≤0.40	4145.64	40.21	
0.41−0.65	4242.51	32.47	0.043
0.66−1.00	4432.52	31.57	< .0001
≥1.01	4538.79	45.11	< .0001
	*Р* for trend		< .0001
Serum insulin (pmol/L)			
≤39.12	4282.89	44.19	
39.13−56.34	4366.38	34.20	0.090
56.35−86.10	4392.92	30.81	0.573
≥86.11	4293.98	48.74	0.0735
	*Р* for trend		0.782

Covariates included age, ethnicity, smoking status, alcohol use, education levels, physical activity and body mass index.

^a^ IGFBP-3: insulin-like growth factor binding proteins-3

^b^ SE: standard error

**Fig 4:**
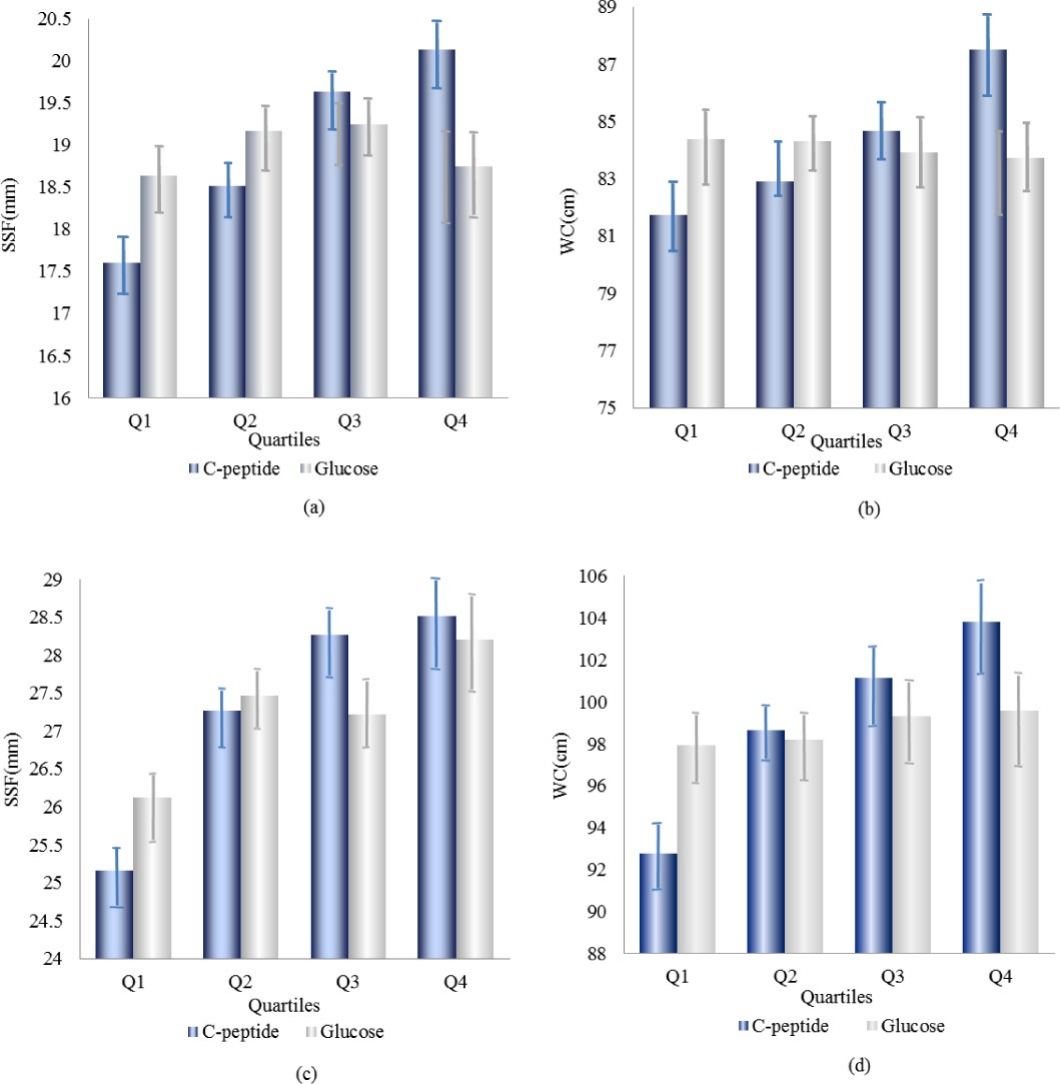
The means of subscapular skinfold and waist circumference in different quartiles of serum C-peptide and plasma glucose by insulin level. The means of subscapular skinfold and waist circumference in different quartiles of serum C-peptide and plasma glucose for insulin level (<56.34 pmol/L) that were presented in (a) and (b), and the means of subscapular skinfold and waist circumference in different quartiles of serum C-peptide and plasma glucose for insulin level (≥56.34 pmol/L) that were presented in (c) and (d).

In Table 4 and Fig 4, the stratification results of the serum insulin levels showed that the serum C-peptide levels were significantly associated with changes in SSF and WC, and a strong linear trend was obtained in each stratification (*P* < .001); however, no significant association was observed between plasma glucose level and changes in SSF and WC.

**Table 4:** The change of subscapular skinfold and waist circumference in different quartiles of serum C-peptide and plasma glucose by stratified analysis for insulin level.

Variables	SSF^a^ (mm)		WC^b^ (cm)	
	Mean	*Р* Value	Mean	*Р* Value
Serum insulin level <56.34 (pmol/L)				
Serum C-peptide level				
Q1^c^	17.61		81.74	
Q2^d^	18.51	0.011	82.92	0.013
Q3^e^	19.63	<0.001	84.68	<0.001
Q4^f^	20.14	<0.001	87.50	<0.001
	*Р* for trend	<0.001	*Р* for trend	<0.001
Plasma glucose level				
Q1	18.64		84.69	
Q2	19.17	0.13	84.32	0.43
Q3	19.25	0.81	83.93	0.41
Q4	18.75	0.30	83.73	0.70
	*Р* for trend	0.769	*Р* for trend	0.09
Serum insulin level ≥56.34 (pmol/L)				
Serum C-peptide level				
Q1	25.16		92.75	
Q2	27.27	<0.001	98.65	<0.001
Q3	28.27	<0.002	101.15	<0.001
Q4	28.52	0.563	103.82	<0.001
	*Р* for trend	<0.001	*Р* for trend	<0.001
Plasma glucose level				
Q1	26.13		97.93	
Q2	27.47	0.001	98.21	0.70
Q3	27.22	0.25	99.32	0.09
Q4	28.21	0.48	99.57	0.65
	*Р* for trend	0.20	*Р* for trend	0.01

Covariates included age, ethnicity, smoking status, alcohol use, education levels, physical activity and body mass index.

^a^SSF: subscapular skinfold

^b^WC: waist circumference

^c^Q1: first quartile,

^d^Q2: second quartile,

^e^Q3: third quartile,

^f^Q4: fourth quartile

## Discussion

The national prospective observational study demonstrated that the serum C-peptide level was associated with increased breast cancer deaths, and further analyses showed that the association was independent of insulin level. The serum C-peptide level was positively associated with the serum IGFBP-3 level, and a significant dose–response association was observed by a linear trend test; however, the association was not obtained with insulin. Moreover, the serum C-peptide levels remained associated with increased SSF and WC after stratified analysis of serum insulin levels.

Few studies have shown that serum C-peptide level is associated with increased risk of breast cancer, and C-peptide has only been assessed as a marker for pancreatic insulin secretion. Moreover, in a previous report, a hypothetical mechanism has been described in which C-peptide acts as a marker of insulin resistance and increases breast cancer incidence. However, recent studies have confirmed that C-peptide is independent of insulin and has an important role in fat distribution and lipid metabolism [16, 17]. The association between obesity and breast cancer has been extensively reported and published. Partly, these results may be attributed to the increased production of estrogen secondary to the aromatase activity in breast adipose tissue; additionally, obesity increases inflammatory cytokines, circulating insulin, and IGF-1 levels [18, 19]. In the present study, we assessed the association between serum C-peptide level and SSF and WC by using stratified analysis for serum insulin level. A strong linear relationship was observed between serum C-peptide level and SSF and WC. Furthermore, previous studies have provided strong evidence and possible explanations for the obtained result. Several studies have found that C-peptide may regulate the expression of peroxisome proliferator-activated receptor-γ (PPARγ)-regulated genes, such as those involved in metabolic control and inflammation; moreover, the ability of C-peptide to activate PPARγ is stronger compared to that of insulin [20]. Studies have revealed that PPARγ is required for the differentiation of adipose tissue as a tissue-specific transcription factor. PPARγ can enhance lipid accumulation in adipocytes, thereby causing lipid metabolism disorders, such as elevated TG levels and reduced high-density lipoprotein cholesterol (HDL-C) levels [21, 22]. The PPARγ has key roles in the regulation of adipogenesis, inflammation, and lipid and glucose metabolism. On the other hand, PPARγ has been studied extensively for its application in potential breast cancer therapies, but the level of PPARγ expression is significantly higher in human breast cancer tissues [23, 24]. Other studies have shown that C-peptide stimulates PPARγ activity in a ligand-independent fashion via PI3-kinase, and C-peptide positively controls the expression of the PPARγ-regulated CD36 scavenger receptors in human THP-1 monocytes. Furthermore, studies have found the high CD36 expression in breast cancer cells [25].

Recent studies have shown that altered cholesterol metabolism, as a comorbidity of obesity, has emerged as an independent risk factor in breast cancer [26]. Multiple studies have shown that low level of HDL-C is associated with increased breast cancer risk [27]. Moreover, few studies have reported that the HDL-C level is inversely associated with IGF-I level [28, 29], and high level of IGF-I is associated with increased risk of breast cancer in pre-menopausal women. However, the mechanism is still unclear. Additionally, studies have shown that low HDL-C level accompanied by obesity may favor increased cellular cholesterol content, and thus produce oxysterol 27-hydroxycholesterol (27HC). 27HC may be the primary biochemical link between lipid metabolism and cancer. Studies have shown that 27HC is an estrogen-receptor agonist in breast cancer cells and stimulates tumor growth and metastasis in several breast cancer models [30]. A more recent study has found that serum C-peptide level is highly negatively associated with HDL-C level, after adjusting for serum insulin level and potential confounding factors. Experimental studies have shown that C-peptide can enhance the release of nitric oxide, thereby resulting in nitrotyrosine production. Nitrotyrosine may induce nitration of HDL-C and produce many oxidized HDL-C, thereby leading to low HDL-C levels. Furthermore, oxidized HDL-C can regulate PPARγ expression via mitogen-activated protein kinase pathways [31]. Recent studies have identified the G protein-coupled receptor 146 as a C-peptide receptor that is highly expressed in adipose tissue and has an important influence on lipid metabolism. Moreover, studies have shown that the copy number variations in the GPR146 gene are associated with many major diseases, including cancer.

IGFBP-3 is known as the main binding partner for circulating insulin-like growth factors and regulates their bioavailability [32]. Several studies have suggested that increased circulating IGFBP-3 levels is associated with increase in BMI and breast cancer risk [33, 34]. Previous epidemiological studies have yielded inconsistent results [35, 36]. Studies have shown that the IGFBP-3 variation in intact or functional versus total IGFBP-3 levels among subjects may modulate the risk of breast cancer in different manners. High levels of intact IGFBP-3 and some IGFBP-3 fragments could be associated with reduced breast cancer risk, whereas high levels of total IGFBP-3 could be associated with increased breast cancer risk [37, 38]. However, recent experimental results have suggested that IGFBP-3 may stimulate adipose tissue expansion and enhance mammary tumor growth, potentially by suppressing T-cell infiltration into tumors. The high expression of IGFBP-3 in breast cancer tumors has also been associated with poor prognosis [39, 40]. Further studies have shown that IGFBP-3 may exert pro-survival or proliferative effects, as well as pro-apoptotic effects, on tumor cells; additionally, studies have demonstrated that IGFBP-3 modulates overall tumor growth [41, 42].

In the present study, we observed that serum C-peptide level was strong positively associated with circulating IGFBP-3 level, and this finding may explain the mechanism by which C-peptide increases breast cancer deaths, at least in part. Overall, the possible mechanisms by which C-peptide affects breast cancer pathogenesis are complex and multifactorial. Our study demonstrates that C-peptide, as an important bioactive peptide, plays a trigger role in the development and growth of breast cancer and it is independent of insulin. Notably, recent update on the Human Gene Database showed that the expression of C-peptide receptor in adipose tissue is much higher than that in other 26 body’s tissues and organs. Recent studies have found that the lack of GPR146 plays an important role in the prevention of hypercholesterolemia. Moreover, the cancer genome database showed that higher expression levels of GPR146 in patients with breast cancer. These findings further support the results of current study [43–45].

The main strength of this study is that it was based upon a nationally prospective follow-up study. The data were collected through a large national surveillance system, and the determination of biomarkers was performed according to standard methodology. We controlled for several important confounding factors by using highly representative biomarker data. Moreover, this is the first study to investigate the association between serum C-peptide level and breast cancer independent of insulin level as a bioactive peptide. The serum insulin level, as an important confounding factor, was controlled using stratified analysis. ANCOVA analyses were conducted to identify the strong linear association between serum C-peptide level and circulating IGFBP-3 level, but serum insulin level did not show significant association with circulating IGFBP-3 level. The ANCOVA result provided strong evidence for some previous etiological assumptions. The study has a major limitation. This is a prospective cohort study and provides new evidence and potential explanations on breast cancer pathogenesis in the population level. If there is a significant association between GPR146 expression levels and breast cancer death or progression, it will strongly support the conclusion that C-peptide itself promote the cancer progression. However, at present, there are no study about the causal link between the GPR146 and breast cancer death. Accordingly, the approaches for evaluating the impact of C peptide on breast cancer pathology and molecular mechanisms are needed for further study. In addition, hyperinsulinemia resulting from insulin resistance is a well-known risk factor for several malignancies, including breast cancer. In this study, no significant association was found between insulin levels and breast cancer mortality after adjusting for confounding factors. Although we used stepwise regression to select the optimal regression equation, the results were obtained on the basis of statistical analysis only. Moreover, C-peptide level is a more sensitive indicator of insulin resistance than insulin level. Therefore, the present result showed that C-peptide is an independent risk factor that increases the risk of breast cancer requires additional supporting evidence.

In conclusion, the present study indicated that serum C-peptide level is associated with increased risk of breast cancer death, and it is independent of insulin level. The partial mechanism may be linked to the changes in increased level of circulating IGFBP-3, and the finding is supported by previous studies. However, the association between circulating IGFBP-3 level and serum insulin level was not observed in the study. Our results suggested that serum C-peptide may increase the risk of breast cancer death via a pathway that is associated with increased IGFBP-3 level.
